# A novel human coronavirus OC43 genotype detected in mainland China

**DOI:** 10.1038/s41426-018-0171-5

**Published:** 2018-10-30

**Authors:** Yun Zhu, Changchong Li, Li Chen, Baoping Xu, Yunlian Zhou, Ling Cao, Yunxiao Shang, Zhou Fu, Aihuan Chen, Li Deng, Yixiao Bao, Yun Sun, Limin Ning, Chunyan Liu, Ju Yin, Zhengde Xie, Kunling Shen

**Affiliations:** 1 Beijing Key Laboratory of Pediatric Respiratory Infection Diseases, Key Laboratory of Major Diseases in Children, Ministry of Education, National Clinical Research Center for Respiratory Diseases, National Key Discipline of Pediatrics (Capital Medical University), Beijing Pediatric Research Institute, Beijing Children’s Hospital, Capital Medical University, National Center for Children’s Health, Beijing, China; 2 The 2nd Affiliated Hospital and Yuying Children’s Hospital of Wenzhou Medical University, Wenzhou, China; 3 Beijing Children’s Hospital, Capital Medical University, National Center for Children’s Health, Beijing, China; 4 The Children’s Hospital-Zhejiang University School of Medicine, Hangzhou, China; 5 Children’s Hospital Capital Institute of Pediatrics, Beijing, China; 6 Shengjing Hospital of China Medical University, Shenyang, China; 7 Children’s Hospital of Chongqing Medical University, Chongqing, China; 8 The First Affiliated Hospital of Guangzhou Medical University, Guangzhou, China; 9 Guangzhou Women and Children’s Medical Center, Guangzhou, China; 10 Xin Hua Hospital Affiliated to Shanghai Jiao Tong University School of Medicine, Shanghai, China; 11 Yinchuan Women and Children Healthcare Hospital, Yinchuan, China; 12 Children’s hospital of Changchun, Changchun, China

Dear Editor,

Coronaviruses (CoVs) have a broad spectrum in humans and other animals, causing asymptomatic infections or respiratory tract infections, gastroenteritis, and neurological diseases of varying severity^1–3^. CoVs are the largest known RNA viruses, belonging to the family Coronaviridae^4^. On the basis of serology and genome phylogeny, CoVs are divided into four genera named *Alpha-*, *Beta-, Delta*-, and *Gamma-coronavirus*^5,6^. To date, six human coronaviruses (HCoVs) have been identified, including two α-CoVs (HCoV-229E and HCoV-NL63) and four β-CoVs (HCoV-OC43, HCoV-HKU1, severe acute respiratory syndrome CoV (SARS-CoV) and Middle East respiratory syndrome CoV(MERS-CoV))^7^. Human coronavirus OC43 (HCoV-OC43) is more predominant than other HCoVs, especially in children and elderly persons^7,8^. In addition to the high nucleotide substitution rates, the genotype shift by natural recombination of HCoV-OC43 is thought to be an adapting mechanism for maintaining its epidemic^8,9^. Since the first isolation of HCoV-OC43 in the 1960s, seven genotypes (A–G) have been identified by phylogenetic analysis of the main genes, such as spike (S), RNA-dependent RNA polymerase (RdRp) and nucleocapsid (N), as well as the whole genome^8–13^. However, the molecular epidemiological data of HCoV-OC43, especially its genotype shift, are scarce in China. Here, we report a novel HCoV-OC43 genotype identified in mainland China.

From November 2014 to November 2016, a prospective study was conducted in hospitalized children with community acquired pneumonia (CAP) at 13 hospitals located in mainland China. A total of 2721 cases were enrolled into this study. The presence of HCoV-OC43 nucleic acid was screened by using an RVP Fast V2 kit (Luminex, USA) with a Luminex Magpix after RNA extraction from throat swabs or nasopharyngeal aspirates. Total RNA from specimens was converted into cDNA using oligo (dT) primers and the SuperScript IV Reverse Transcription System (Invitrogen, Carlsbad, CA). The full-length S, RdRp, N, and viral genomes (from the 5′-end of the ORF1a gene to the 3′-end of the poly-A tail) were amplified from HCoV-OC43-positive samples by a genome walking method involving a total of 44 overlapping fragments using a set of specific primers (Supplementary Table S1)^8,12^. The genome sequence was determined as previously described^8^. Sequences were aligned using the ClustalW program implemented in MEGA 5.03 (version 5.0; Sudhir Kumar, Arizona State University, Tempe, AZ, USA). Maximum likelihood (ML) trees of whole-genome sequences and the full-length sequences of the S, RdRp, and N genes were constructed with the best-fit general time reversible model with gamma-distributed rate variation across sites and 1000 bootstrap replicates implemented in MEGA 5.03^14^. Neighbor-joining trees of 24 known genes and whole genomes were constructed with Kimura’s two-parameter model and 1000 bootstrap pseudoreplicates implemented in MEGA 5.03^14^. To analyze potential recombination events, the complete genome sequences of HCoV-OC43 were aligned and analyzed using the similarity plot and boot scanning method in Simplot (version 3.5.1, http://sray.med.som.jhmi.edu/SCRoftware). All sequences generated in this study have been deposited in GenBank, and the accession numbers are MG197709 to MG197723 (Supplementary Table S2). The reference sequences were retrieved from GenBank on December 2017.

HCoV-OC43 was detected in 1.5% (42/2721) of enrolled cases. A total of 15 whole genomes of HCoV-OC43 were obtained from 42 respiratory specimens of OC43-positive cases. To identify the genotype of OC43-positive samples, the ML phylogenetic trees based on the full-length sequences of the S, RdRp, and N genes were constructed by using the representative strains of genotypes A–G (Supplementary Fig. S1). Phylogenetic analysis of the S gene clustered all reference strains in genotypes A–G, which agreed with previous reports^8,10–13^. The 15 OC43 strains identified in the present study were organized into two clusters (Supplementary Fig. 1A). Eight OC43 strains, including HZ-459/16, BJ-165/15, BJ-124/15, BJ-221/15, GZFE-26/15, YC-207/15, WZ-522/15, and WZ-303/15, clustered into genotype G (D-like) lineage, with a high nucleotide similarity (99.0–99.9%) to strains isolated in Malaysia. The other seven strains (BJ-112/15, BJ-164/15, YC-72/15, YC-55/15, YC-68/15, YC-67/15, and CC-23/15) fell into the genotype B lineage and shared nucleotide similarity (96.0–99.7%) with strains identified in Beijing. However, all the 15 strains from the present study had close relationships with the representative strains of genotype D or G (D-like) in the ML tree of the RdRp gene (Supplementary Fig. 1B). Importantly, the bootstrap values at several nodes, such as genotype D or C, were lower than 70% in the phylogenetic tree of the RdRp gene, which led to an unresolved tree. The possible reasons for this result maybe due to the highly conserved nucleotides compared to the other genes and less genetic information in GenBank. Analysis of N genes showed that eight strains belonged to genotype G strains in the tree of the S genes clustered together, while the other strains belonged to genotype B strains in the tree of S genes clustered together (Supplementary Fig. 1C). The incongruence of several phylogenetic analyses of different genes suggested the occurrence of recombination.

To further explore the evolutionary characteristics of the 15 OC43 strains, a ML tree was generated using the whole-genome sequences and was compared to other whole genomes of OC43 strains deposited in GenBank. These reference strains were divided into genotypes from A to G as reported by Oong et al.^13^. Eight OC43 strains clustered with genotype G strains circulating in Malaysia with high nucleotide similarity (99.2–99.6%). However, the other seven OC43 strains clustered into a dependent novel lineage (Fig. 1a). Based on the estimation of the intergenotype pairwise genetic distances, the distances of the novel lineage compared with genotypes B, C, D, F, and G were <0.7%, but the distances were >0.9% when compared with genotypes A and E (Fig. 1b). These results suggested that the novel lineage had a closer evolutionary relationship with genotypes B, C, D, F, and G. Genotype D was the descendant of the recombination events between genotypes B and C. Genotypes G and F were both D-like genotypes, which showed similar recombination patterns in most parts of the sequence with genotype D strains, except for parts of the nsp10 gene. The lowest whole-genome-sequence genetic distance between distinct genotypes (A–G) of HCoV-OC43 was 0.26 ± 0.02% (between genotypes F and D) in a previous study^13^. According to these criteria, the mean distances of a novel lineage compared with the other seven identified genotypes ranged from 0.45 ± 0.02% to 0.99 ± 0.01%, which suggested that a novel genotype of HCoV-OC43 emerged. The novel genotype was designated as genotype H. To further analyze the recombination structures of genotype H strains, neighbor-joining trees of 24 known genes and whole-genome sequences were constructed. Eighteen whole-genome sequences of HCoV-OC43 strains, which belonged to genotypes A–G, were used as reference strains (Supplementary Fig. S2). The seven genotype H strains showed a close relationship with the reference strains belonging to genotypes D, G (D-like), and F (D-like) in the phylogenetic trees of most nonstructural protein genes (nsp1–nsp16), the NS2a gene, and the HE gene. However, the NS4, E, M, N, I and whole-genome sequences were clustered with the genotype B strains, and the S gene showed a close relationship with genotype B and E strains. Subsequently, we constructed a similarity plot and performed boot scanning analysis using full-length genome sequences. From the 5′-end of the genome to position 23,000 nt, genotype H strains showed a greater similarity to genotype F (D-like) strains. From positions 23,000 nt to 27,000 nt, genotype H strains were closely related to genotype E strains. From positions 27,000 nt to the 3′-end of the genome, genotype H was closely related to genotype B. These findings were consistent with the phylogenetic analysis of the 24 genes and suggested that the occurrence of natural recombination events resulted in the emergence of the novel genotype H of HCoV-OC43 (Supplementary Fig. S3A and S3B). The demographic and clinical profiles of children infected with HCoV-OC43 genotype G or Hin the present study are summarized in Table 1.

**Fig. 1:**
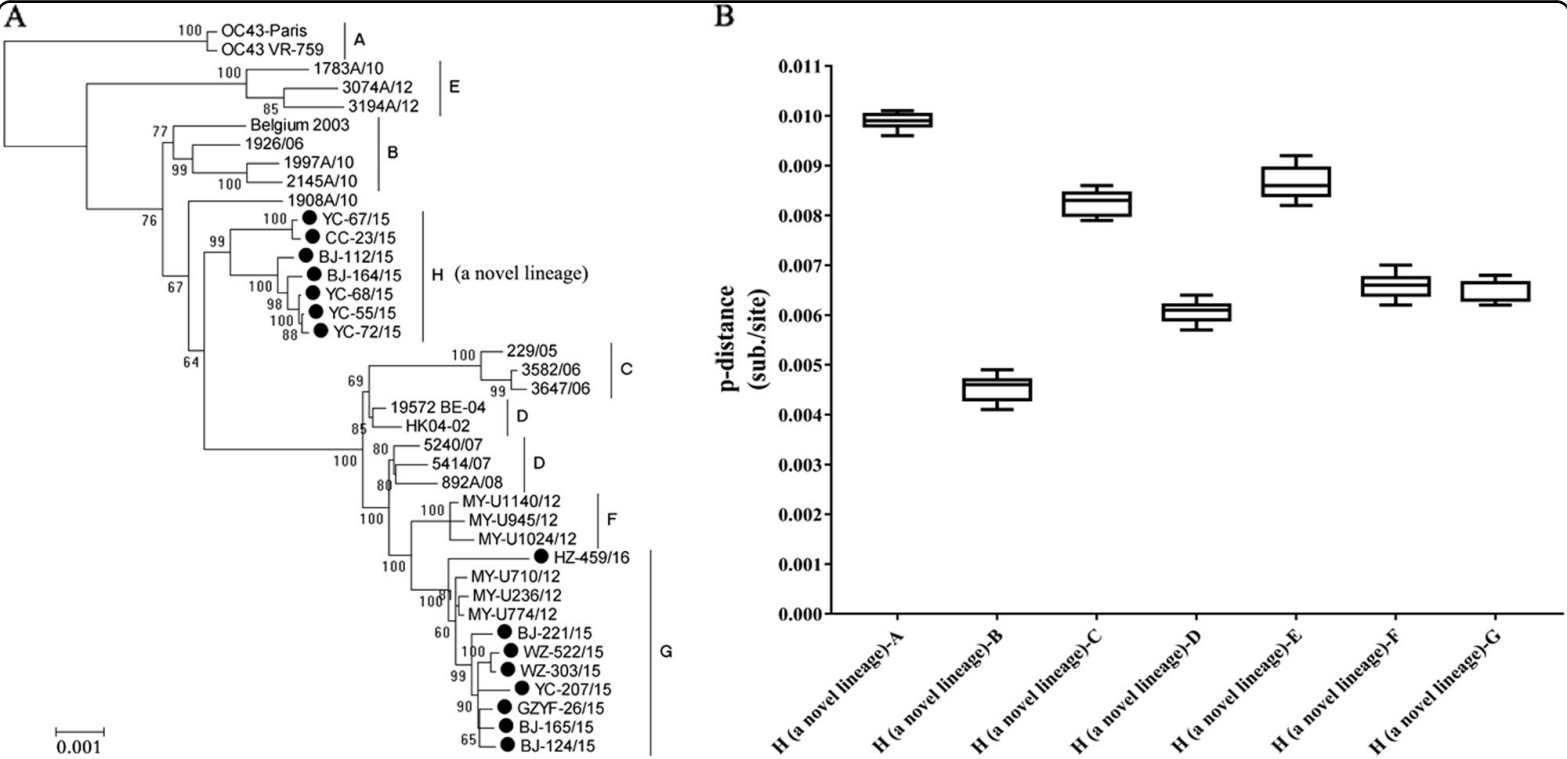
Genetic analysis of the HCoV-OC43 strains based on the whole-genome sequences. **a** Phylogenetic tree of the HCoV-OC43 strains based on whole-genome sequences. The tree was constructed by using the maximum likelihood (ML) method with the best-fit general time reversible model with gamma-distributed rate variation across sites and 1000 bootstrap replicates implemented in MEGA 5.03. Bootstrap values over 70% are shown in the nodes. **b** Estimation of pairwise genetic distances between genotype H and genotypes A–G of HCoV-OC43 strains based on the whole-genome sequences

**Table 1:** Demographic and clinical manifestation of HCoV-OC43 positive cases

Genotype	Strains ID	Collection date	Age (year)	Gender	Clinical manifestation
G	BJ-124/15	2015/5/6	0.08	Male	Cough, Sneezing, nasal discharge
	WZ-303/15	2015/5/6	1.58	Male	Fever, cough, expectoration
	WZ-522/15	2015/5/16	6.58	Male	Cough, nasal discharge
	GZYF-26/15	2015/5/18	3.83	Male	Fever, cough, nasal discharge, headache, sore throat
	BJ-165/15	2015/6/9	0.42	Male	Fever, cough, expectoration
	BJ-221/15	2015/8/14	1.17	Male	Fever, expectoration
	YC-207/16	2016/5/5	2.75	Male	Fever, expectoration
	ZJ-459/16^a^	2016/6/20	9.25	Female	Fever, pectoralgia, pleural effussion
H	YC-55/15	2015/3/12	3.25	Female	Fever, cough, expectoration
	BJ-112/15	2015/4/22	0.17	Female	Fever, expectoration
	BJ-164/15	2015/6/2	0.5	Female	Cough, sore throat, hoarseness of voice
	YC-67/15	2015/6/4	0.83	Female	Fever, cough
	YC-68/15	2015/6/5	0.42	Male	Fever, expectoration
	YC-72/15	2015/6/13	4.92	Male	Fever
	CC-23/15	2015/7/13	2.33	Female	Fever, expectoration

Severe pneumonia case was indicated by symbol “a”

In summary, the present study reported a novel HCoV-OC43 recombinant genotype H, which was detected among children with CAP in mainland China. The novel genotype H might have been generated by recombination events among putative parental genotype D-like, genotype E, and genotype B strains. Our results emphasize the need for continuous surveillance of HCoV-OC43 in mainland China to better understand the mechanisms of the phylo dynamics of HCoV-OC43.

## Electronic supplementary material

Supplementary Table S1

Supplementary Table S2

Supplementary Figure S1

Supplementary Figure S2

Supplementary Figure S3
