# Supplementary material for: A novel human coronavirus OC43 genotype detected in mainland China
Source: Emerg Microbes Infect. 2018 Oct 30;7:173. doi: 10.1038/s41426-018-0171-5 (PMC6207742; doi:10.1038/s41426-018-0171-5)
Supplement: Supplementary file 1 — Supplementary Table S1 [file 41426_2018_171_MOESM1_ESM.docx]

**Supplementary Table S1** Primers used for full-length genome sequencing of HCoV-OC43

| **Primer name** | **Primer sequence (5'-3')** | **Target gene** | **Reference** |
| --- | --- | --- | --- |
| OC43F65 | CATCCACTCCCTGTAATCTAT | ORF1a | [1] |
| OC43R665 | AACTGATAGGCCACATGCTTA | ORF1a | [1] |
| OC43F557 | ATGCTATGTGAGAGGTTGTAATCC | ORF1a | [2] |
| OC43R1531 | TATGCAATACAGGGTTAACAGG | ORF1a | [2] |
| OC43F1410 | GGTTGGATTCCTGGTAACATGA | ORF1a | [2] |
| OC43R2431 | TTTTCTGCCTGAAAGACAAATTC | ORF1a | [2] |
| OC43F2317 | TTGCACAAGCATTTCAGAGTGTT | ORF1a | [2] |
| OC43R3396 | GCACATACAGACCCAAAACTTTAAC | ORF1a | [2] |
| OC43F3279 | GATGTAGAAATGTCGGATTTTG | ORF1a | [2] |
| OC43R4405 | TAAAGACACATCAGAAGGCA | ORF1a | [2] |
| OC43F4293 | AGAACACAGGGTAAACAAAGTT | ORF1a | [2] |
| OC43R5523 | CAAGATCTTCACGACTCAATGT | ORF1a | [2] |
| OC43F5450 | TTGCATGTAAATGTGGTGTAA | ORF1a | [1] |
| OC43R6033 | ATCAAAACCCAACTTAGCATTA | ORF1a | [1] |
| OC43F5949 | CGAGAAAGTAGATGGTGTGTAT | ORF1a | [1] |
| OC43R6614 | CGCACAGCTTTAACCACATT | ORF1a | [1] |
| OC43F6426 | TGATGTGTATGATATGTGGCTTA | ORF1a | [1] |
| OC43R6934 | TAATCCACTGTGCAATCTTA | ORF1a | [1] |
| OC43F6472 | TGAGCAGAGCAGTTAACGTACCTA | ORF1a | [2] |
| OC43R7177 | TGGATAAAACCATGCCGTATACA | ORF1a | [2] |
| OC43F7090 | GAAGCTGATAGGAGAGCATT | ORF1a | [1] |
| OC43R8204 | AAATATGTTGGCACCAAGTTA | ORF1a | [1] |
| OC43F8068 | CGTAAAAGTTGTTCTATTGATTCAG | ORF1a | [2] |
| OC43R8400 | TCTGCTTATTATAAGTAAGCTTCAG | ORF1a | [2] |
| OC43F8304 | TATATGGTCTGTGGATGCTTT | ORF1a | [1] |
| OC43R8910 | AAGCACACAGCCACTAGCAT | ORF1a | [1] |
| OC43F8775 | CCCTACCAAAGTGTTACGATA | ORF1a | [1] |
| OC43R9427 | CACACCACACAATCACGTTA | ORF1a | [1] |
| OC43F9314 | CTGGTGCTATACTCGCTGTAA | ORF1a | [1] |
| OC43R9925 | AAGCAGTAGGCGGTTGGTAA | ORF1a | [1] |
| OC43F9844 | GCTTGCTCTCAGTTGGCTAA | ORF1a | [1] |
| OC43R10484 | CCATTGAAGTCAGTACCAGTAT | ORF1a | [1] |
| OC43F10297 | AAACCACAAGGAGCCTTTCATGTAA | ORF1a | [2] |
| OC43R10780 | CTACCCATAATCTGACGTCCTTGG | ORF1a | [2] |
| OC43F10656 | CAATGGATTTAGCCAAGTTA | ORF1a | [1] |
| OC43R11435 | ACAGCAACCCACTTAGCAAT | ORF1a | [1] |
| OC43SF11302 | GGTACAAGGGTTCTAACTTAGAGG | ORF1a | [2] |
| OC43SR12124 | TAAGCAGATTTAGCAATATTACAGG | ORF1a | [2] |
| OC43F12021 | CGTTGAATATGAAGTTGCTAA | ORF1a | [1] |
| OC43R12439 | CCGCATAGGTAACATAGACATT | ORF1a | [1] |
| OC43F12318 | TGTGTACCATTGAATGCAATA | ORF1a | [2] |
| OC43R12804 | GACCTTTAGCATCTTGAACAG | ORF1a | [2] |
| OC43F12703 | CTTAGTGATGTTGATGGTCTTA | ORF1a | [1] |
| OC43R13285 | AACTTCCATCCCGCCAAAAT | ORF1a | [1] |
| OC43F13180 | AGTTTGTACAAGTGCCTGTAGGT | RdRp | [2] |
| OC43R14301 | TCCACAAAAATTTGCCTAACAAG | RdRp | [2] |
| OC43F14160 | GGAGTATGCCATATCATCCTAACAC | RdRp | [2] |
| OC43R15162 | CAATACGTAGTAGGTTTGGCATAGC | RdRp | [2] |
| LPW 3064F | CTGGGATGATATGTTACGCCG | RdRp | [1] |
| LPW 2579R | GTGTGTTGTGAACARAAYTCRTG | RdRp | [1] |
| LPW 1223F | TAAGTGCCTTTCAACAGGT | RdRp | [1] |
| LPW 1127R | KGCCTTTTGCGTTTCTGC | RdRp | [1] |
| OC43F16382 | ACAGGATCTCCGTACATAGACGA | ORF1b | [2] |
| OC43R17471 | TTTCATAAACCAAGGCGGAC | ORF1b | [2] |
| OC43F17315 | GCACCACGTGTGTTATTGAG | ORF1b | [2] |
| OC43R18345 | TTAAATTGTTCACCAGGAGGA | ORF1b | [2] |
| OC43F18174 | GTGCTCATGCCACGCTTGATA | ORF1b | [2] |
| OC43R19271 | TAGCATCCATACCATCCATATACACACA | ORF1b | [2] |
| OC43F19059 | CACCGAATGCAGTTGTATGTAGAT | ORF1b | [2] |
| OC43R20157 | TAACACGGCTTTGTGTAAAGATAG | ORF1b | [2] |
| OC43F19994 | GTGCGTAAAGAAGGTCAGGATGT | ORF1b | [2] |
| OC43R20879 | GAATAGTACCAGCAGGCAACC | ORF1b | [2] |
| OC43F20689 | GAAGCCAGTTACTTTGCCTACAG | ORF1b | [2] |
| OC43R21691 | TTTATAGTCTTCAGGCTGTATGTCC | ORF1b | [2] |
| OC43F21507 | ATGCAGACAAGCCTAATCATTTTAT | NS2α | [2] |
| OC43R22266 | CGGAAATGAAAACTTTTCCTGTAG | NS3α | [2] |
| OC43HE22158F | AGCTAGAAGAAGGCGATCTTCC | HE | [2] |
| OC43HE23056R | TGAGACCATAAATAACACCAGTGTC | HE | [2] |
| OC43HE22867F | CCTGCTTATATAGCTCCTCAAGC | HE | [2] |
| OC43HE23770R | TCAGTACTTATAGAAGGAGGACCG | HE | [2] |
| LPW 1162F | CCYRTTTGTRTGTATGATCC | S | [1] |
| LPW 1166R | YGCATAAAAAGTACCACC | S | [1] |
| LPW 1261F | CTRCTATARYTATAGGTAGT | S | [1] |
| LPW 2094R | GCCCAAATTACCCAATTGTAGG | S | [1] |
| LPW 2095F | TGATGCTGCTAAGATATATGG | S | [1] |
| LPW 2098R | ATTCCGARATAGCAATGCTGG | S | [1] |
| LPW 1839F | ATCTTTTGTATGATTCTAATGG | S | [1] |
| LPW 1178R | GACACCAAGMCCATTAAT | S | [1] |
| OC43F26457 | GACCTCATTTGTGTGCAAAG | S | [2] |
| OC43R27323 | GAAGGTTGGGTATTGAAGTG | S | [2] |
| OC43F27204 | ATACTTGGATGTACACTGGTAGTG | S | [2] |
| OC43R27806 | ATATAACGGAGAAATTTCTTCTCA | S | [2] |
| OC43F27758 | TCCAATCTAGCATTTGTTACCACG | NS5α, E, M | [2] |
| OC43R28982 | AACAGCAAAACCACTAGTATCGCTT | NS5α, E, M | [2] |
| OC43F28865 | ACTAGGTATTGGCTATTCTTTGGC | M, N | [2] |
| OC43R29830 | GCATCCTTGCCAAGTTTTGC | M, N | [2] |
| OC43F29639 | GGGTTACTATATTGAAGGCTCAGG | N | [2] |
| OC43R30658 | ATGCTGGCTCTTCCCTTTG | N | [2] |
| 3’raceF | AATGGATGTCTTGCTGCTAT |  | [1] |
| 3’raceR-1 | GACCACGCGTATCGATGTCGACTTTTTTTTTTTTTTTTV |  | [1] |
| 3’raceF-2 | GTATAGTGTTGGAGAAAGTGAA |  | [1] |
| 3’raceR-2 | GACCACGCGTATCGATGTCGAC |  | [1] |
| 5’raceR392 | TTTAAGAAGTCGGCGACAAT |  | [1] |
| 5’raceR152 | TCCCTGGCTGAAAGCTGTTA |  | [1] |

1. Lau, S.K., et al. Molecular epidemiology of human coronavirus OC43 reveals evolution of different genotypes over time and recent emergence of a novel genotype due to natural recombination. J. Virol. 85,11325-11337 (2011).
2. Zhang, Y., et al. Genotype shift in human coronavirus OC43 and emergence of a novel genotype by natural recombination. J. Infect. 70, 641-650 (2014).
