# Supplementary material for: A novel human coronavirus OC43 genotype detected in mainland China
Source: Emerg Microbes Infect. 2018 Oct 30;7:173. doi: 10.1038/s41426-018-0171-5 (PMC6207742; doi:10.1038/s41426-018-0171-5)
Supplement: Supplementary file 2 — Supplementary Table S2 [file 41426_2018_171_MOESM2_ESM.docx]

**Supplementary Table S2 Background information of the HCoV-OC43 gene sequences used in this study**

| **Strain name** | **Accession NO.** | **Sampling year** | **Sampling sites** | **Gene** | **Reference** |
| --- | --- | --- | --- | --- | --- |
| BJ-112/15 | MG197709 | 2015 | Beijing,China | Complete Genome | This study |
| BJ-124/15 | MG197710 | 2015 | Beijing,China | Complete Genome | This study |
| BJ-164/15 | MG197711 | 2015 | Beijing,China | Complete Genome | This study |
| BJ-165/15 | MG197712 | 2015 | Beijing,China | Complete Genome | This study |
| BJ-221/15 | MG197713 | 2015 | Beijing,China | Complete Genome | This study |
| CC-23/15 | MG197714 | 2015 | Changchun,China | Complete Genome | This study |
| GZYF-26/15 | MG197715 | 2015 | Guan zhou,China | Complete Genome | This study |
| WZ-303/15 | MG197716 | 2015 | We zhou, China | Complete Genome | This study |
| WZ-522/15 | MG197717 | 2015 | Wenzhou, China | Complete Genome | This study |
| YC-55/15 | MG197718 | 2015 | Yinchuan, China | Complete Genome | This study |
| YC-67/15 | MG197719 | 2015 | Yinchuan, China | Complete Genome | This study |
| YC-68/15 | MG197720 | 2015 | Yinchuan, China | Complete Genome | This study |
| YC-72/15 | MG197721 | 2015 | Yinchuan, China | Complete Genome | This study |
| YC-207/15 | MG197722 | 2015 | Yinchuan, China | Complete Genome | This study |
| ZJ-459/16 | MG197723 | 2016 | Zhejiang, China | Complete Genome | This study |
| OC43-Paris | AY585229 | 1967 | Paris, France | Complete Genome | (1) |
| OC43VR-759 | AY585228 | 1967 | USA | Complete Genome | (1) |
| Belgium 2003 | AY903459 | 2003 | Belgium | Complete Genome | (2) |
| 19572 BE-04 | AY903460 | 2004 | Belgium | Complete Genome | (2) |
| HK04-01 | JN129834 | 2004 | Hong Kong, China | Complete Genome | (3) |
| HK04-02 | JN129835 | 2004 | Hong Kong, China | Complete Genome | (3) |
| MY-U236/12 | KX538966 | 2012 | Kuala Lumpu, Malaysia | Complete Genome | (4) |
| MY-U710/12 | KX538970 | 2012 | Kuala Lumpu, Malaysia | Complete Genome | (4) |
| MY-U774/12 | KX538970 | 2012 | Kuala Lumpu, Malaysia | Complete Genome | (4) |
| MY-U945/12 | KX538974 | 2012 | Kuala Lumpu, Malaysia | Complete Genome | (4) |
| MY-U1140/12 | KX538977 | 2012 | Kuala Lumpu, Malaysia | Complete Genome | (4) |
| MY-U1024/12 | KX538975 | 2012 | Kuala Lumpu, Malaysia | Complete Genome | (4) |
| 229/05 | KF572816 | 2005 | Beijing, China | S | (5) |
| 1926/06 | KF572807 | 2006 | Beijing, China | S | (5) |
| 3582/06 | KF572817 | 2006 | Beijing, China | S | (5) |
| 3647/06 | KF572818 | 2006 | Beijing, China | S | (5) |
| 5240/07 | KF572844 | 2007 | Beijing, China | S | (5) |
| 5414/07 | KF572849 | 2007 | Beijing, China | S | (5) |
| 892A/08 | KF572868 | 2008 | Henan, China | S | (5) |
| 1783A/10 | KF572804 | 2010 | Beijing, China | S | (5) |
| 1908A/10 | KF572805 | 2010 | Beijing, China | S | (5) |
| 1997A/10 | KF572808 | 2010 | Neimenggu, China | S | (5) |
| 2145A/10 | KF572810 | 2010 | Shandong, China | S | (5) |
| 3074A/12 | KF572812 | 2012 | Hebei, China | S | (5) |
| 3194A/12 | KF572814 | 2012 | Henan, China | S | (5) |
| 229/05 | KF572907 | 2005 | Beijing, China | RdRp | (5) |
| 1926/06 | KF572900 | 2006 | Beijing, China | RdRp | (5) |
| 3582/06 | KF572913 | 2006 | Beijing, China | RdRp | (5) |
| 3647/06 | KF572914 | 2006 | Beijing, China | RdRp | (5) |
| 5240/07 | KF572918 | 2007 | Beijing, China | RdRp | (5) |
| 5414/07 | KF572923 | 2007 | Beijing, China | RdRp | (5) |
| 892A/08 | KF572943 | 2008 | Henan, China | RdRp | (5) |
| 1783A/10 | KF572897 | 2010 | Beijing, China | RdRp | (5) |
| 1908A/10 | KF572898 | 2010 | Beijing, China | RdRp | (5) |
| 1997A/10 | KF572901 | 2010 | Neimenggu, China | RdRp | (5) |
| 2145A/10 | KF572904 | 2010 | Shandong, China | RdRp | (5) |
| 3074A/12 | KF572908 | 2012 | Hebei, China | RdRp | (5) |
| 3194A/12 | KF572911 | 2012 | Henan, China | RdRp | (5) |
| 229/05 | KF572753 | 2005 | Beijing, China | N | (5) |
| 1926/06 | KF572742 | 2006 | Beijing, China | N | (5) |
| 3582/06 | KF572758 | 2006 | Beijing, China | N | (5) |
| 3647/06 | KF572760 | 2006 | Beijing, China | N | (5) |
| 5240/07 | KF572764 | 2007 | Beijing, China | N | (5) |
| 5414/07 | KF572769 | 2007 | Beijing, China | N | (5) |
| 892A/08 | KF572797 | 2008 | Henan, China | N | (5) |
| 1783A/10 | KF572739 | 2010 | Beijing, China | N | (5) |
| 1908A/10 | KF572740 | 2010 | Beijing, China | N | (5) |
| 1997A/10 | KF572744 | 2010 | Neimenggu, China | N | (5) |
| 2145A/10 | KF572750 | 2010 | Shandong, China | N | (5) |
| 3074A/12 | KF572746 | 2012 | Hebei, China | N | (5) |
| 3194A/12 | KF572748 | 2010 | Henan, China | N | (5) |

1. St-Jean, J.R., et al. Human respiratory coronavirus OC43: genetic stability and neuroinvasion. J. Virol. 78, 8824-8834 (2004).
2. Vijgen L., et al. Circulation of genetically distinct contemporary human coronavirus OC43 strains. Virology 337, 85-92 (2005).
3. Lau, S.K., et al. Molecular epidemiology of human coronavirus OC43 reveals evolution of different genotypes over time and recent emergence of a novel genotype due to natural recombination. J. Virol. 85, 11325-11337 (2011).
4. Oong, X.Y., et al. Identification and evolutionary dynamics of two novel human coronavirus OC43 genotypes associated with acute respiratory infections: phylogenetic, spatiotemporal and transmission network analyses. Emerg. Microbes infect. 6,e3 (2017).
5. Zhang, Y., et al. Genotype shift in human coronavirus OC43 and emergence of a novel genotype by natural recombination. J. Infect. 70, 641-650 (2014).
