# Supplementary figures and images for: A novel human coronavirus OC43 genotype detected in mainland China
Source: Emerg Microbes Infect. 2018 Oct 30;7:173. doi: 10.1038/s41426-018-0171-5 (PMC6207742; doi:10.1038/s41426-018-0171-5)

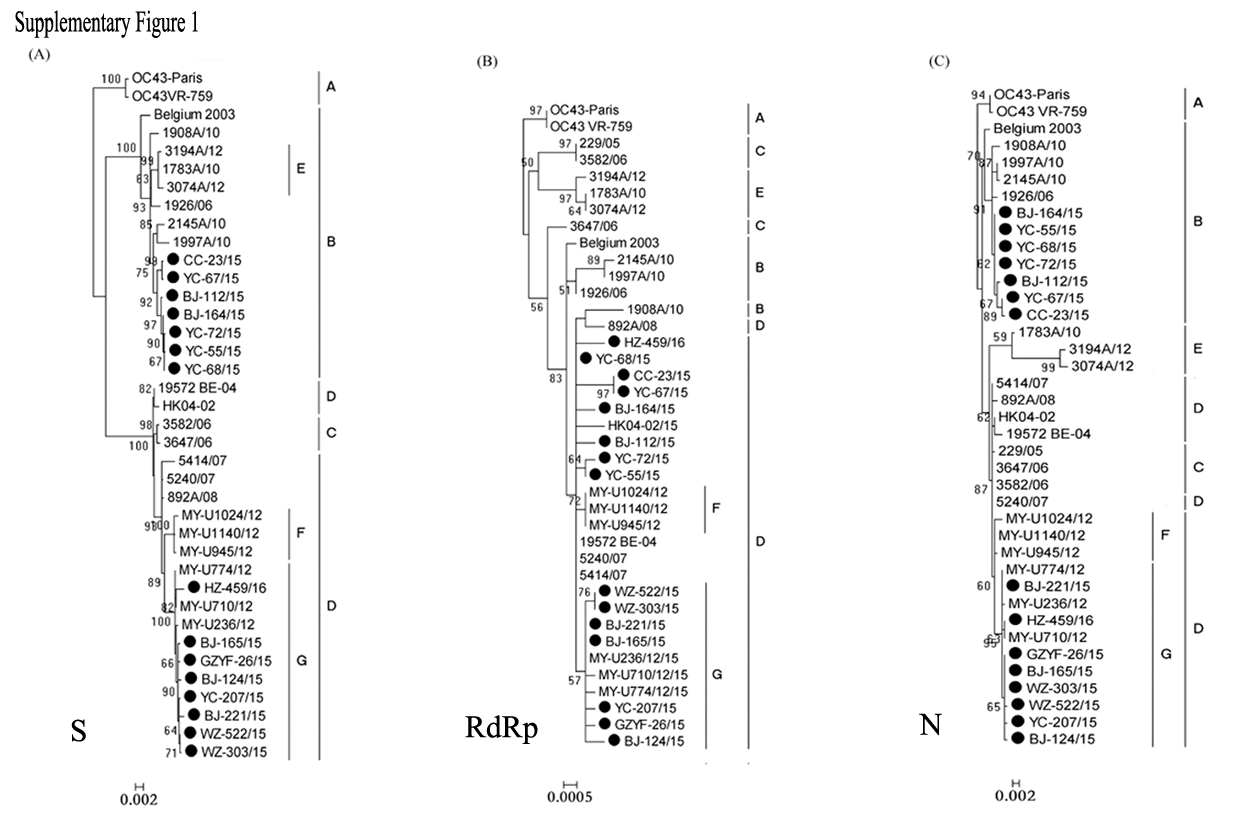

Supplement: Supplementary file 3 — Supplementary Figure S1 [file 41426_2018_171_MOESM3_ESM.tif]

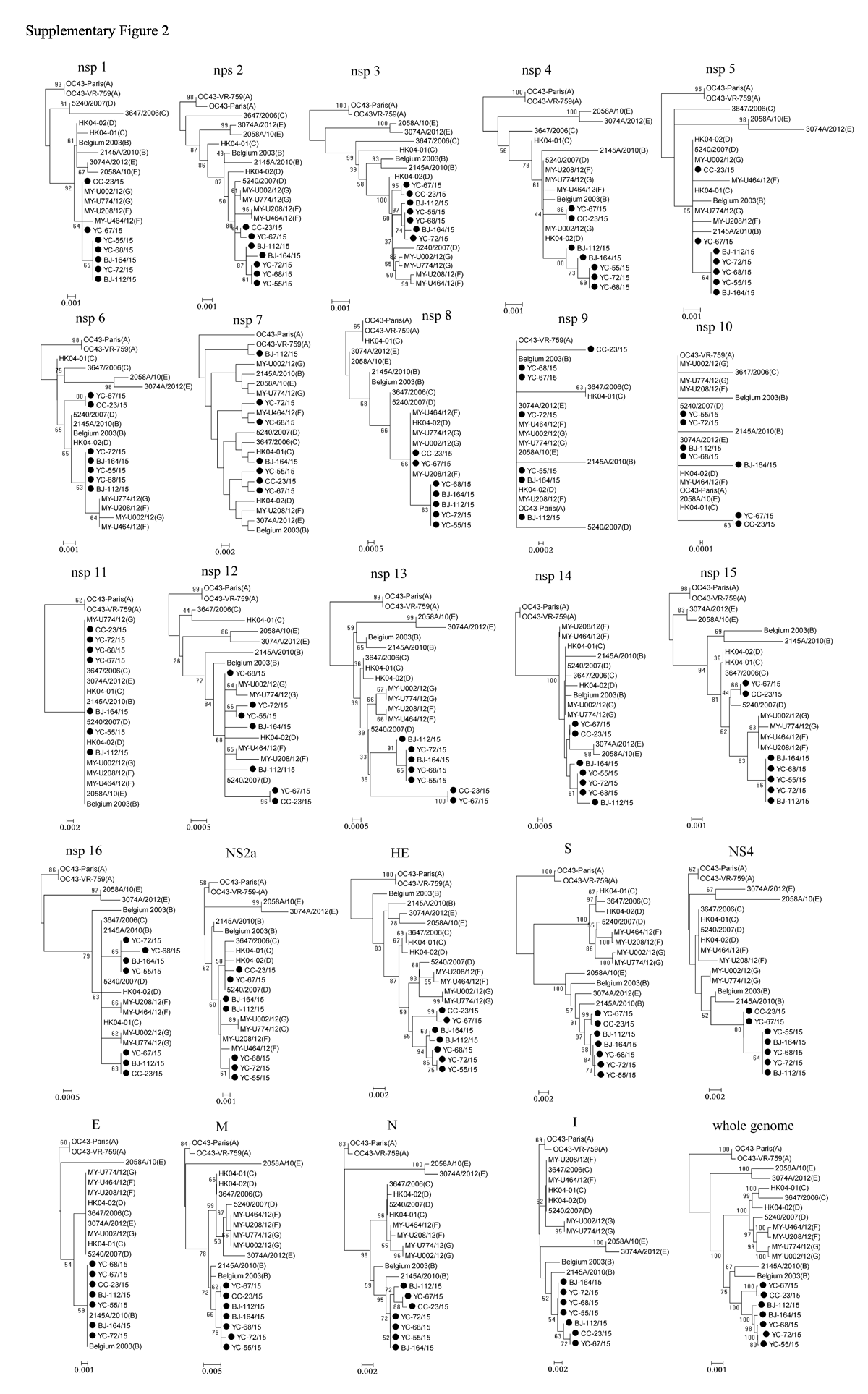

Supplement: Supplementary file 4 — Supplementary Figure S2 [file 41426_2018_171_MOESM4_ESM.tif]

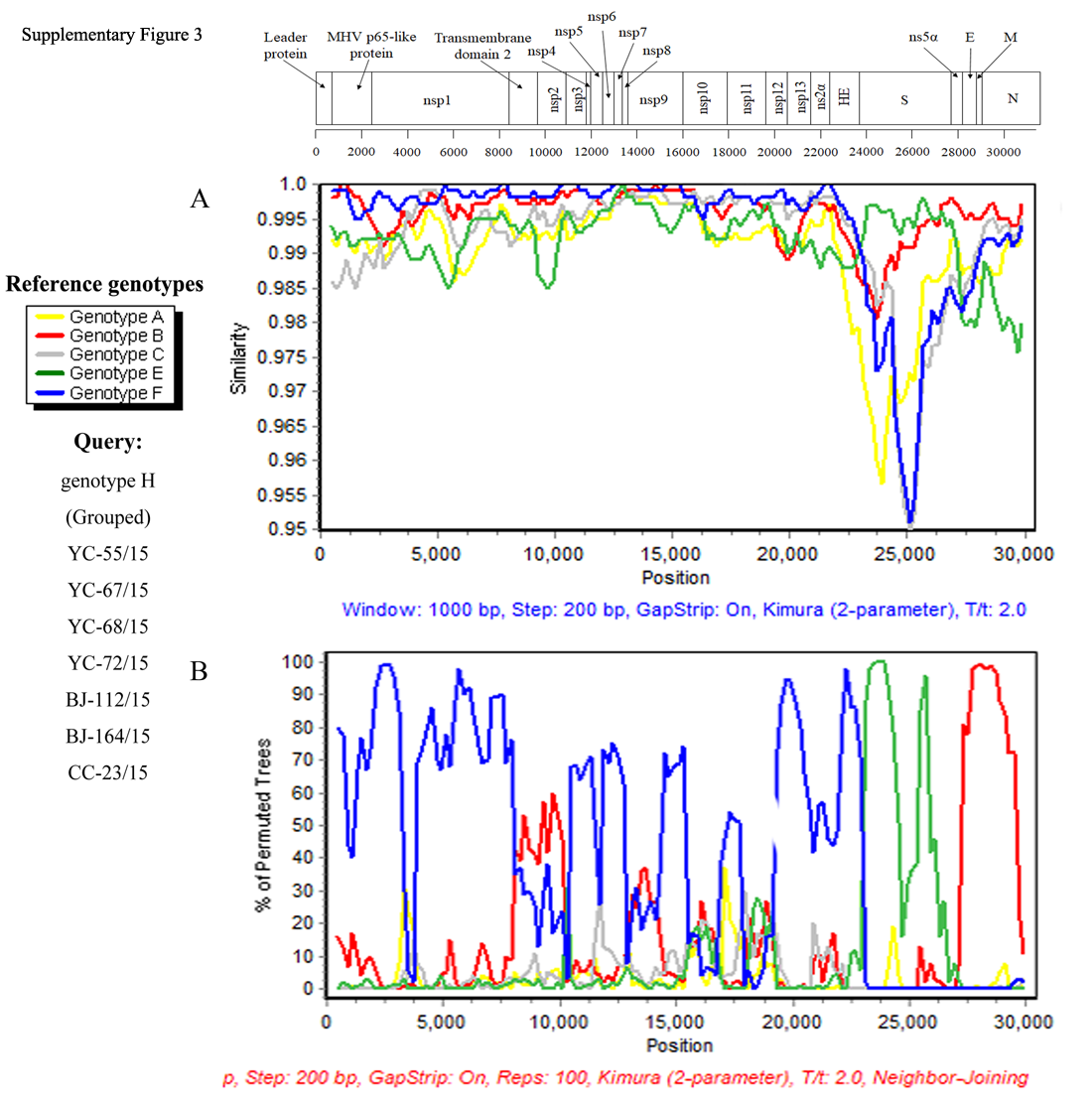

Supplement: Supplementary file 5 — Supplementary Figure S3 [file 41426_2018_171_MOESM5_ESM.tif]
